# Left atrial longitudinal strain as a predictor of Cancer therapeutics-related cardiac dysfunction in patients with breast Cancer

**DOI:** 10.1186/s12947-020-00210-5

**Published:** 2020-07-21

**Authors:** Hyukjin Park, Kye Hun Kim, Hyung Yoon Kim, Jae Yeong Cho, Hyun Ju Yoon, Young Joon Hong, Hyung Wook Park, Ju Han Kim, Youngkeun Ahn, Myung Ho Jeong, Jeong Gwan Cho

**Affiliations:** Department of Cardiology, Chonnam National University Medical School/Hospital, 42 Jaebongro, Dong-gu, Gwangju, 61469 South Korea

**Keywords:** Left atrium, Strain, Chemotherapy, Echocardiography

## Abstract

**Background:**

We investigated the usefulness of the left atrial (LA) strain measurement on the prediction of upcoming cancer therapeutics-related cardiac dysfunction (CTRCD) after trastuzumab therapy in patients with breast cancer who did not develop CTRCD after chemotherapy.

**Methods:**

A total of 72 females with breast cancer who did not develop CTRCD after chemotherapy and underwent additional trastuzumab therapy were divided into CTRCD (*n* = 13) and no CTRCD group (*n* = 59). Echocardiographic measurements including left ventricular global longitudinal strain (LVGLS) and peak atrial longitudinal strain (PALS) decline were compared.

**Results:**

CTRCD was identified in 13 patients (18.1%) after additional trastuzumab therapy. Baseline echocardiographic findings were not different. After the completion of chemotherapy, conventional echocardiographic parameters were not different, but PALS decline (15.0 ± 4.7 vs. 8.9 ± 3.2%, *p* < 0.001) and LVGLS decline (10.5 ± 1.3 vs. 9.1 ± 1.1%, *p* = 0.002) were significantly greater in CTRCD than in no CTRCD group. PALS decline at the time of chemotherapy completion could predict future CTRCD after trastuzumab therapy with better sensitivity and specificity (cutoff value 11.79%, sensitivity 76.9% and specificity 81.4%) than LVGLS decline (cutoff value 9.9%, sensitivity 69.2% and specificity 78.0%).

**Conclusions:**

PALS or LVGLS decline developed before developing overt CTRCD after chemotherapy for breast cancer, and PALS decline showed better sensitivity and specificity in predicting future CTRCD than LVGLS decline. Serial measurement of PALS can be used as a useful parameter in the prediction of future CTRCD.

## Introduction

With the improvements in early detection and therapeutic advances including chemotherapy, cancer survival rates have been gradually improved [1]. In contrary to these improvement in cancer survival, cancer therapeutics-related cardiac dysfunction (CTRCD) became a major concern, and it affects considerable portion of patients who underwent chemotherapy. Various chemotherapeutic agents, have been reported to have higher risk of CTRCD [2], and patients with human epidermal growth factor receptor II (HER2)-positive breast cancer are particularly at high risk, since many of them receive anthracycline, anti-microtubule agents, and trastuzumab-based chemotherapy with or without radiation therapy, which are all known to have high risk of CTRCD. Echocardiography is the most widely used imaging modality in the evaluation of CTRCD, for its wide availability, no exposure to radiation or contrast agents, relatively low cost, and its ability to evaluate hemodynamic state of the heart. Conventionally, left ventricular ejection fraction (LVEF) has been broadly used as a diagnostic criterion [2], and recent studies have suggested that LV global longitudinal strain (GLS) is a useful parameter in detecting subclinical myocardial damage and thus can be used as a predictor of upcoming CTRCD [3]. The usefulness of other echocardiographic parameters such as isovolumic relaxation time, peak early velocity (E), peak atrial velocity (A) of pulsed wave mitral doppler flow, E/A ratio, E deceleration time [4, 5] or early diastolic mitral annular velocity (e’) were also evaluated in the prediction of upcoming CTRCD, but these parameters have not shown consistent clinical significance or usefulness to predict upcoming CTRCD.

Recently, left atrial (LA) GLS using 2-dimensional (2D) speckle-tracking echocardiography has been suggested as a method to represent LA functional state. It is a new promising approach for LA mechanics and electromechanical coupling [6], and it showed good correlation with pulmonary capillary wedge pressure, even better than E/e’ ratio in advanced heart failure (HF) [7]. However, the clinical significance of LA strain measurement in the prediction of upcoming CTRCD in patients with breast cancer has been poorly studied. Therefore, the authors investigated the usefulness of peak LA longitudinal strain (PALS) in the prediction of upcoming CTRCD after additional trastuzumab therapy in HER2-positive breast cancer patients who did not develop CTRCD after chemotherapy.

## Materials and methods

### Study design and population

The present study is a single-center retrospective observational study and the study protocol was approved by the institutional review board of our center (IRB No. 2015–05-092).

**Fig. 1:**
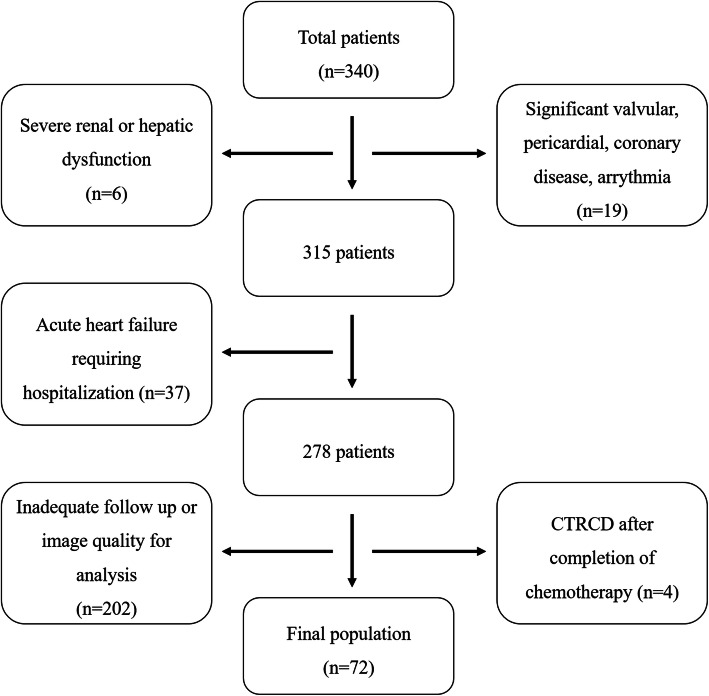
Flow chart for the inclusion of study population. CTRCD, cancer therapeutics-related cardiac dysfunction

From January 2015 to January 2017, among 753 female patients with breast cancer, a total of 340 patients who finished adjuvant target therapy with trastuzumab after the completion of chemotherapy were identified. After excluding 268 patients, a total of 72 patients who did not develop CTRCD after chemotherapy and underwent additional trastuzumab therapy with baseline, post-chemotherapy, and post-trastuzumab echocardiographic examinations were enrolled and divided into 2 groups; CTRCD group (*n* = 13, 47.2 ± 7.7 years) versus no CTRCD group (*n* = 59, 49.2 ± 8.8 years). CTRCD was defined as a decrease in LVEF > 10% from the baseline LVEF to an absolute value below 55% on follow up echocardiography [2] after the initiation of trastuzumab therapy in the present study. The reasons of exclusion were as follows; significant valvular, pericardial, coronary heart diseases, and arrhythmia (*n* = 19), severe renal or hepatic dysfunction (*n* = 6), acute heart failure (HF) requiring hospitalization (*n* = 37), inadequate echocardiographic follow up or inappropriate echocardiographic images for strain analysis (*n* = 202), development of CTRCD after completion of chemotherapy (*n* = 4) (Fig. 1).

### Echocardiographic examination

Echocardiographic examinations were performed at least 3 times in all patients. Baseline echocardiographic examination was done before performing surgery or initiating chemotherapy in all patients. First follow up echocardiography was performed just after completing all scheduled cycles of chemotherapy and before initiating trastuzumab therapy. Second follow up echocardiography was performed just after the completion of trastuzumab therapy. In cases of symptoms or signs of HF, however, second follow-up echocardiography was performed as soon as possible, even though all the scheduled cycles of trastuzumab therapy were not completed.

Echocardiographic examination was performed at resting state, in the left lateral decubitus position, by 2 trained sonographers and 2 digital echocardiographic equipment systems (Vivid E9 and E95, Vingmed Ultrasound, Horten, Norway), according to the current guidelines and diagnostic criteria by American Society of Echocardiography and European Association of Cardiovascular Imaging [8]. All echocardiographic examinations were performed at the day when the patient was free of intravenous fluid, to minimize effect of the patient’s fluid status on cardiac function and structure. Digital cine images and still images were acquired and stored for subsequent offline analysis.

LVGLS was calculated from apical 3-, 4-, and 2-chamber views by 2D speckle tracking methods using automated functional imaging. LA GLS was also calculated by 2D speckle tracking techniques using a commercially available 2D echocardiography analysis software (EchoPac [GE Vingmed Ultrasound, Horten, Norway]), from apical 4- and 2-chamber views. The LA border was manually defined, divided into 6 segments, and traced. The region of interest was adjusted to match with LA thickness. LA GLS value was calculated by averaging values of all 12 segments (6 from apical 4- and 6 from apical 2-chamber view). LA reservoir function was evaluated by PALS, during relaxation of the LA. Simultaneously, LA conduit and booster strain were measured in the LA GLS curve. When 2 or more segments were not able to be adequately tracked in LVGLS or LA GLS, the case was excluded from the analysis. Volume of the LA was checked using the Simpson’s method from apical 4- and 2- chamber views during end-systole [9–11]. A total of 516 echocardiography examinations were checked and analyzed for 129 patients. PALS and LVGLS were successfully checked in 433 (83.9%) and 472 (91.5%) examinations, respectively. Since a patient was excluded in the analysis when PALS or LVGLS was not feasible in any of 4 serial echocardiography examinations in each patient, finally 72 (55.8%) patients were included in the analysis. In all cases, LVEF was measured according to the modified biplane Simpson’s method. Other conventional measurements were done for all echocardiography cases, and all values including GLS values were measured and calculated 3 times and were averaged by 1 cardiologist, who was blinded to other information about each patient.

Echocardiographic parameters including LVGLS and PALS decline were compared between the groups. LVGLS and PALS decline were calculated by the following formula; [(LVGLS or PALS at baseline – LVGLS or PALS at the time of chemotherapy completion) / LVGLS or PALS at baseline].

### Statistical analyses

Statistical Package for the Social Sciences (SPSS) 22.0 for Microsoft Windows (SPSS, Inc., Chicago, IL, USA) was used for all statistical analyses. All numerical variables are presented as mean value ± standard deviation and were compared by independent samples t-test or one-way analysis of variance. All categorical variables were shown as their number and percentages, and they were compared using Chi-square or Fisher’s exact test to determine the significance of differences. Receiver operation characteristic (ROC) curve analysis was used to determine optimal cutoff values, sensitivity and specificity of PALS to predict upcoming CTRCD, and to compare them with those of LVGLS. All statistical tests were two-tailed, and a *p* value < 0.05 was considered as a significant one.

## Results

### Baseline characteristics

CTRCD was not identified on follow up echocardiography which was performed after the completion of chemotherapy, but identified in 13 patients (18.1%) on echocardiography which was performed during (7 [9.7%] patients) or just after completing trastuzumab therapy (6 [8.3%] patients).

Baseline characteristics are summarized in Table 1. Among patients who received doxorubicin, significantly higher dose was administered in CTRCD group. Otherwise, there were no significant differences in baseline characteristics between the groups.

**Table 1:** Baseline characteristics

	CTRCD (*n* = 13)	No CTRCD (n = 59)	*p* value
Age (years)	47.2 ± 7.7	49.2 ± 8.8	0.471
Hypertension (%)	2 (15.4)	10 (16.9)	1.000
ACEi or ARB use (%)	1 (7.7)	6 (10.2)	0.629
Beta-blocker use (%)	0 (0.0)	3 (5.1)	0.545
Diabetes mellitus (%)	1 (7.7)	4 (6.8)	1.000
Dyslipidemia (%)	2 (15.4)	5 (8.5)	0.602
Smoking (%)	1 (7.7)	2 (3.4)	0.455
BMI (kg/m^2^)	24.9 ± 4.5	23.8 ± 3.9	0.412
Epirubicin (%)	5 (38.5)	29 (49.2)	0.485
Epirubicin dose (mg/m^2^)	336.0 ± 68.4	314.5 ± 88.4	0.610
Doxorubicin (%)	5 (38.5)	16 (27.1)	0.504
Doxorubicin dose (mg/m^2^)	304.8 ± 38.3	266.6 ± 32.0	0.038
Cyclophosphamide (%)	8 (61.5)	38 (64.4)	1.000
Cyclophosphamide dose (mg/m^2^)	2550.0 ± 424.3	2460.0 ± 222.8	0.388
Docetaxel (%)	4 (30.8)	19 (32.2)	1.000
Docetaxel dose (mg/m^2^)	337.5 ± 75.0	331.6 ± 150.7	0.940
Radiation therapy (%)	7 (53.8)	27 (45.8)	0.597
Radiation dose (cGy)	5754.9 ± 398.8	5628.4 ± 362.3	0.426

CTRCD: cancer therapeutics-related cardiac dysfunction, ACEi: angiotensin converting enzyme inhibitor, ARB: angiotensin receptor blocker, BMI: body mass index

### Echocardiographic findings

Baseline echocardiographic findings are summarized in Table 2. Baseline echocardiographic findings were not different between the groups.

**Table 2:** Baseline echocardiographic findings

	CTRCD (*n* = 13)	No CTRCD (*n* = 59)	*p* value
LVEDVI (mL/m^2^)	56.8 ± 10.6	56.5 ± 9.9	0.920
LVESVI (mL/m^2^)	19.9 ± 5.3	20.3 ± 4.6	0.774
LVEF (%)	65.0 ± 6.4	63.9 ± 5.9	0.564
LVGLS (%)	−21.0 ± 2.4	−22.2 ± 2.2	0.086
E (cm/sec)	71.3 ± 10.4	62.7 ± 7.7	0.001
e’ (cm/sec)	8.8 ± 1.4	8.3 ± 1.8	0.379
s’ (cm/sec)	7.9 ± 0.9	7.4 ± 1.0	0.138
E/e’	8.2 ± 1.0	7.7 ± 1.2	0.201
LAVI (mL/m^2^)	25.8 ± 3.8	26.0 ± 3.9	0.833
PALS (%)	33.1 ± 5.9	33.8 ± 5.6	0.689
LA conduit strain (%)	19.2 ± 3.4	19.8 ± 3.3	0.550
LA booster strain (%)	13.9 ± 2.5	14.0 ± 2.3	0.905
RVSP (mmHg)	29.3 ± 7.1	32.0 ± 7.3	0.239

*CTRCD* cancer therapeutics-related cardiac dysfunction, *LVEDVI* left ventricular end-diastolic volume index, *LVESVI* left ventricular end-systolic volume index, *LVEF* left ventricular ejection fraction, *LVGLS* left ventricular global longitudinal strain, *E* early diastolic mitral inflow velocity, e’: early diastolic velocity of mitral septal annulus, s’: systolic velocity of mitral septal annulus, *LAVI* left atrial volume index, *PALS* peak atrial longitudinal strain, *LA* left atrium, *RVSP* right ventricular systolic pressure

Serial changes of echocardiographic findings in CTRCD group are summarized in Table 3. After the completion of chemotherapy, conventional echocardiographic parameters except for e’ velocity were not changed, but LVGLS and PALS were significantly decreased. After the completion of trastuzumab therapy, LV and LA volumes were significantly increased, whereas LVEF were significantly decreased. Diastolic functional parameters were also significantly impaired. LVGLS and PALS were significantly further decreased as compared to those at the time of chemotherapy completion.

**Table 3:** Changes of the echocardiographic findings in patients with cancer therapeutics-related cardiac dysfunction

	Baseline	After chemotherapy	After trastuzumab therapy
LVEDVI (mL/m^2^)	56.8 ± 10.6	57.1 ± 12.3	65.7 ± 12.0
LVESVI (mL/m^2^)	19.9 ± 5.3	20.4 ± 6.7	35.0 ± 7.7†‡
LVEF (%)	65.0 ± 6.4	64.5 ± 7.4	46.9 ± 4.8†‡
LVGLS (%)	−21.0 ± 2.4	− 18.8 ± 2.3*	− 15.3 ± 2.6†‡
E (cm/sec)	71.3 ± 10.4	67.5 ± 7.8	84.3 ± 14.7†‡
e’ (cm/sec)	8.8 ± 1.4	7.6 ± 1.3*	7.0 ± 0.8‡
s’ (cm/sec)	7.9 ± 0.9	7.3 ± 1.3	4.5 ± 1.0†‡
E/e’	8.2 ± 1.0	9.0 ± 1.0	12.0 ± 1.4†‡
LAVI (mL/m^2^)	25.8 ± 3.8	25.8 ± 3.6	29.1 ± 3.8†‡
PALS (%)	33.1 ± 5.9	28.2 ± 5.5*	23.9 ± 6.0†‡
LA conduit strain (%)	19.2 ± 3.4	14.9 ± 2.9*	12.7 ± 3.2‡
LA booster strain (%)	13.9 ± 2.5	13.3 ± 2.7	11.2 ± 2.8‡
RVSP (mmHg)	29.3 ± 7.1	27.1 ± 7.2	30.2 ± 7.9

*LVEDVI* left ventricular end-diastolic volume index, *LVESVI* left ventricular end-systolic volume index, *LVEF* left ventricular ejection fraction, *LVGLS* left ventricular global longitudinal strain, E: early diastolic mitral inflow velocity, e’: early diastolic velocity of mitral septal annulus, s’: systolic velocity of mitral septal annulus, *LAVI* left atrial volume index, *PALS* peak atrial longitudinal strain, *LA* left atrium, *RVSP* right ventricular systolic pressure. *: *p* < 0.05 between baseline and after chemotherapy, †: *p* < 0.05 between after chemotherapy and after trastuzumab therapy, ‡: *p* < 0.05 between baseline and after trastuzumab therapy

Serial changes of echocardiographic findings in no CTRCD group are summarized in Table 4. Conventional echocardiographic findings including LVEF were not changed on follow up echocardiography not only at the time of chemotherapy completion, but also at the time of trastuzumab therapy completion. However, LVGLS and PALS were significantly decreased after the completion of chemotherapy, and LVGLS and PALS were significantly further decreased as compared to those at the time of chemotherapy completion.

**Table 4:** Changes of the echocardiographic findings in patients without cancer therapeutics-related cardiac dysfunction

	Baseline	After chemotherapy	After trastuzumab therapy
LVEDVI (mL/m^2^)	56.5 ± 9.9	55.9 ± 10.2	55.7 ± 10.3
LVESVI (mL/m^2^)	20.3 ± 4.6	20.0 ± 5.1	20.2 ± 5.7
LVEF (%)	63.9 ± 5.9	64.2 ± 6.7	63.8 ± 7.3
LVGLS (%)	−22.2 ± 2.2	−20.2 ± 2.2*	−17.8 ± 2.3†‡
E (cm/sec)	62.7 ± 7.7	61.4 ± 11.6	59.2 ± 11.8
e’ (cm/sec)	8.3 ± 1.8	8.3 ± 1.7	7.9 ± 1.7
s’ (cm/sec)	7.4 ± 1.0	7.3 ± 1.0	7.3 ± 1.1
E/e’	7.7 ± 1.2	7.6 ± 1.5	7.7 ± 1.8
LAVI (mL/m^2^)	26.0 ± 3.9	26.1 ± 4.1	26.7 ± 4.0
PALS (%)	33.8 ± 5.6	30.9 ± 5.7*	28.0 ± 6.0†‡
LA conduit strain (%)	19.8 ± 3.3	16.6 ± 3.0*	15.0 ± 3.2†‡
LA booster strain (%)	14.0 ± 2.3	14.3 ± 2.7	13.0 ± 2.8†‡
RVSP (mmHg)	32.0 ± 7.3	30.8 ± 7.7	29.2 ± 8.1

*LVEDVI* left ventricular end-diastolic volume index, *LVESVI* left ventricular end-systolic volume index, *LVEF* left ventricular ejection fraction, *LVGLS* left ventricular global longitudinal strain, E: early diastolic mitral inflow velocity, e’: early diastolic velocity of mitral septal annulus, s’: systolic velocity of mitral septal annulus, *LAVI* left atrial volume index, *PALS* peak atrial longitudinal strain, *LA* left atrium, *RVSP* right ventricular systolic pressure. *: *p* < 0.05 between baseline and after chemotherapy, †: *p* < 0.05 between after chemotherapy and after trastuzumab therapy, ‡: *p* < 0.05 between baseline and after trastuzumab therapy

**Fig. 2:**
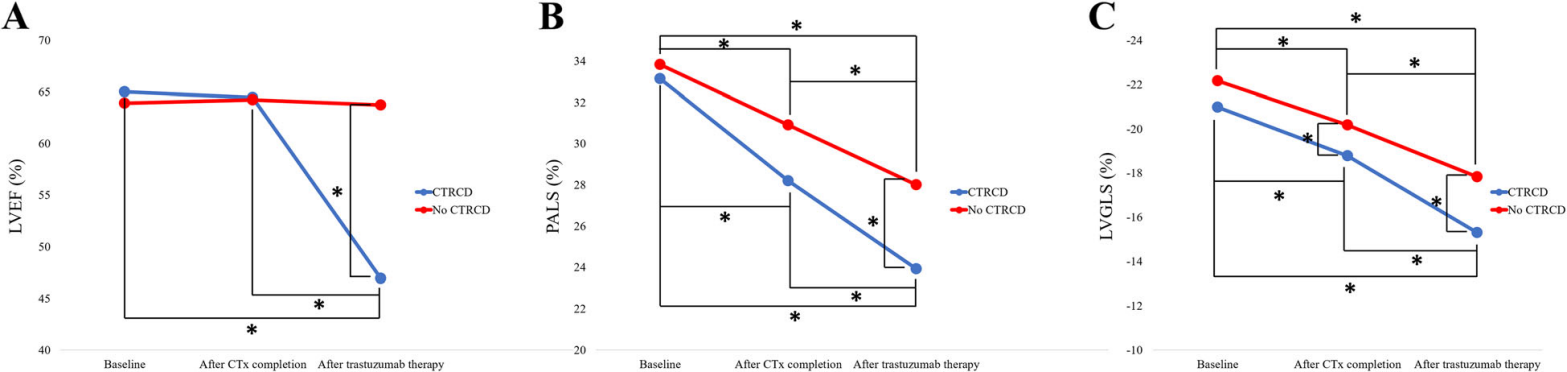
Serial changes in left ventricular ejection fraction (LVEF) (panel A), peak left atrial longitudinal strain (PALS) (panel B), left ventricular global longitudinal strain (LVGLS) in cancer therapeutics-related cardiac dysfunction (CTRCD) group (blue line) and no CTRCD group (red line). CTx, chemotherapy. Statistically significant differences are marked by asterisks (*)

Serial changes of LVEF, LVGLS, and PALS are shown in Fig. 2. LVEF was not changed after the completion of chemotherapy in both CTRCD and no CTRCD group. LVGLS and PALS were significantly decreased after the completion of chemotherapy and significantly further decreased after the completion of trastuzumab therapy not only in CTRCD group, but also in no CTRCD group. However, LVGLS decline (10.5 ± 1.3 vs. 9.1 ± 1.1%, *p* = 0.002) and PALS decline (15.0 ± 4.7 vs. 8.9 ± 3.2%, *p* < 0.001) were significantly greater in CTRCD group than in no CTRCD group. Significant drop (> 15% drop compared with baseline value) of LVGLS occurred in 11 patients (84.6%) in CTRCD group, and 25 patients (42.4%) in no CTRCD group.

### ROC curve analysis

**Fig. 3:**
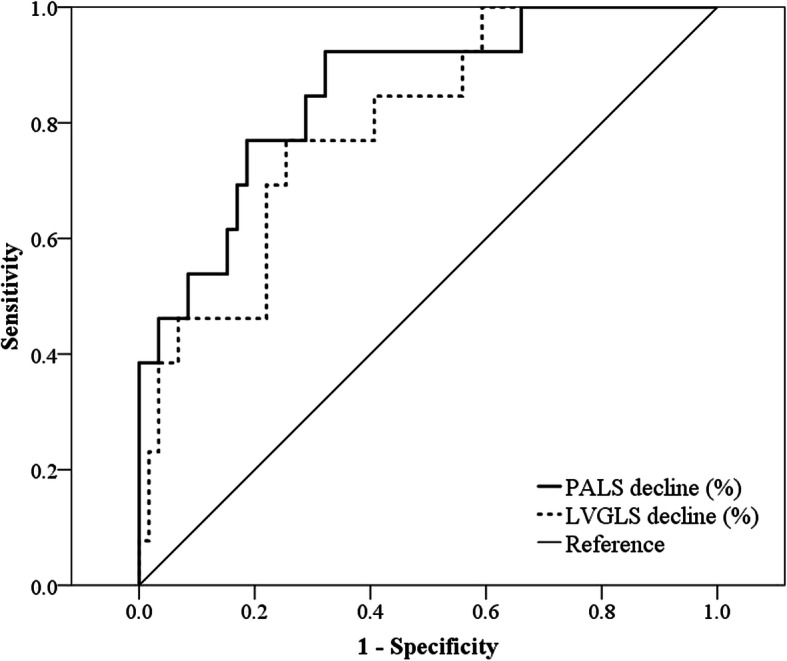
Receiver operation characteristic curves for decline of peak left atrial longitudinal strain (PALS) (solid line) and decline of left ventricular global longitudinal strain (LVGLS) (dotted line) to predict upcoming cancer therapeutics-related cardiac dysfunction

To identify the optimal cut-off value and area under the curve (AUC) for the prediction of future CTRCD, ROC curve analysis was performed. The optimal value to predict future CTRCD was 11.79% for PALS decline and 9.9% for LVGLS. PALS decline at the time of chemotherapy completion could predict future CTRCD after trastuzumab therapy with better sensitivity and specificity (sensitivity 76.9% and specificity 81.4%, area under curve [AUC] 0.854) than LVGLS decline (sensitivity 69.2% and specificity 78.0%, AUC 0.797) (Fig. 3).

## Discussion

The authors investigated the clinical significance of LA strain analysis in the prediction of future CTRCD after additional trastuzumab therapy in patients with HER2-positive breast cancer who did not develop CTRCD after standard chemotherapy, and the present study demonstrated several clinically important findings. First, CTRCD was not uncommon (18.1%) after additional trastuzumab therapy, even in patients who did not develop CTRCD after chemotherapy for breast cancer. Second, after the completion of chemotherapy, conventional echocardiographic parameters were not changed, but PALS and LVGLS were significantly decreased. Therefore, it is suggested that PALS and/or LVGLS changes can be used as a more sensitive marker for cardiac injury after chemotherapy. Third, PALS decline after chemotherapy could predict upcoming CTRCD with better sensitivity and specificity than LVGLS. Therefore, serial measurement of PALS can be used as a useful parameter in the prediction of future CTRCD in patients with breast cancer.

Anthracycline, cyclophosphamide, taxane, and trastuzumab-based chemotherapy is an important and highly effective chemotherapeutic regimen in HER2-positive breast cancer patients, both increasing survival duration of metastatic breast cancer and decreasing cancer recurrence and death after proper surgical treatment [12]. However, both agents carry a significant risk of cardiotoxicity: about 15–30% of patients treated with an anthracycline and trastuzumab are reported to experience CTRCD, and the risk is especially high when anthracycline and trastuzumab are administered concurrently [13, 14].

To date, the most widely accepted diagnostic criterion of CTRCD is based on LVEF. But its utility for early detection or prediction of CTRCD is somewhat limited by its relatively low reproducibility [3], and relatively low sensitivity to detect subclinical cardiotoxicity [4]. As LVGLS is well known to be able to detect subclinical LV dysfunction before evident LV dysfunction develops, recently LVGLS has been used to define and predict future CTRCD [10, 15]. LVGLS has shown a good sensitivity (65–86%) and a good specificity (73–95%) for predicting subsequent declines in LVEF or HF [16], and the finding was reproduced in our results.

Diastolic dysfunction and increase in LV filling pressure occur in earlier stages of various forms of HF. Comprehensive evaluation of diastolic function, using conventional parameters is also recommended, and some previous studies reported early changes of diastolic functional parameters [17, 18]. However, in a more recent study, e’ value showed only insignificant changes between patients with CTRCD and ones without it [19], and similar tendency was observed in our results. Also, until today, none of conventional parameters of diastolic function have been proven to be well correlated with further LV dysfunction. But in the present study, parameters which are known to better reflect diastolic dysfunction and increased in LV filling pressure, such as E/e’ ratio and changes in PALS showed significant impairment in CTRCD group, suggesting presence of diastolic dysfunction in hearts affected by CTRCD. To our best knowledge the present study is the first one to demonstrate it, and also to demonstrate value of PALS in prediction of CTRCD. As LVGLS does, PALS can comprehensively analyze global myocardial function of the LA, and it may reflect impairment of mechanical function of LA muscle before general structural changes of the LA such as LA enlargement by chronic elevation of LV filling pressure occurs.

In this study, the patients who did not experience CTRCD also experienced continuous impairment of PALS, although degree of the change was smaller than the patients with CTRCD. In some previous reports, impairment of LVGLS was observed in patients undergoing breast cancer treatment with trastuzumab, suggesting presence of subclinical cardiac dysfunction in those patients [20]. Our results can be suggesting ‘more subclinical and earlier’ LV dysfunction and subsequent LA dysfunction, or can be suggesting myocardial dysfunction of the LA itself in breast cancer patients undergoing chemotherapy and/or HER2-targeted therapy, irrespective of presence of evident CTRCD. Whether impairment of PALS is only secondary to LV dysfunction, or due to LA myocardial dysfunction itself should be defined in further studies. But until today, we believe impairment of PALS is not a single change and is related to subclinical LV dysfunction and subsequent diastolic dysfunction, since other studies have shown its close correlation with LV filling pressure [7] and in our results PALS shown significant negative correlation with E/e’ ratio, which is already known to be a good surrogate marker of LV filling pressure.

One possible explanation about why diastolic parameters have not shown consistent and serial changes in cardio-oncology setting to date is because many patients with cancer suffer from disability to have enough oral intake, and they are frequently under a lot of fluid therapy, especially for chemotherapy. We tried to minimize their effect in this study, by checking echocardiography when the patients were at the outpatient setting and free of any intravenous fluid therapy at least for the day of echocardiography. Also, presence of ‘gray zone’ in prediction of LV filling pressure [7] could have masked subtle changes of diastolic function in cancer patients, which could be revealed with other fine modalities such as PALS. And as volumetric analyses of the LA can be easily confounded by measurement errors, conventional parameters of diastolic function might not reflect subclinical changes in diastolic function and the LA function, in patients undergoing chemotherapy.

In many other forms of chronic and progressive HF, LA volume is not necessarily normalized despite reduction of LV filling pressure, by irreversible damage and remodeling process. However, PALS seems to be improved by reduction of LV filling pressure [21], suggesting potential role of PALS as a more sensitive monitoring tool of diastolic dysfunction, and a predictive factor of further LV dysfunction as we demonstrated in this study. Still, we do not know whether impaired PALS is restored before or after reverse remodeling and functional recovery of the LV with cessation of trastuzumab and/or guideline-directed HF management. Although whether LV functional recovery is truly reversible or not is still controversial, myocardial dysfunction by trastuzumab is generally considered as a reversible one (type II cardiotoxicity) and global LV function is frequently improved after cessation of trastuzumab. And it is anticipated that we could observe further changes in both LA and LV function and define prognostic utility of PALS and reversibility of LA structure and function with cessation of trastuzumab and medical HF management.

This study has some limitations. First, as shown in Fig. 1, many patients were excluded in the analysis because of inadequate echocardiography follow up schedule because of retrospective nature of this study. Second, some patients were also excluded for poor speckle tracking in LVGLS or in LA GLS. Measuring LA GLS by speckle tracking has several technical limitations: far field location of the LA, dropping-out of several portions (especially the interatrial septum) of the LA wall, and thin LA wall thickness makes it difficult to precisely track the LA. Third, although reference values are being reported recently [10, 22], general application is not so easy yet because of intervendor variabilities and absence of standardized measurement method: to our best knowledge, the vast majority of studies using LA GLS required manual definition of LA borders and region of interest, limiting wide use of it in real practice. Fourth, though we tried to minimize effect of volume loading condition on strain parameters by checking echocardiography when the patients were free of intravenous fluid therapy, such effect could not be fully eliminated when other loading conditions (dehydration, excessive salt and water intake, etc) are present, without other informations such as serum pro-brain natriuretic peptide level. And, since this study is a single center study, our results need to be validated in multicenter studies later.

## Conclusion

In conclusion, this study demonstrated that functional changes of the LA as measured by PALS and functional changes of the LV as measured by LVGLS precede before developing overt CTRCD defined by changes of LVEF. Also, serial changes of PALS showed even better sensitivity and specificity, compared with that of LVGLS. Therefore, the serial measurement of PALS can be used as a useful parameter in the prediction of future CTRCD. As serial changes of the LA in patients undergoing chemotherapy is not clearly defined yet, reversibility of LA structure and function with cessation of trastuzumab and medical HF management should be defined, and further studies are required.

## Data Availability

Not applicable.

## Abbreviations

CTRCD: Cancer therapeutics-related cardiac dysfunction
HER2: Human epidermal growth factor receptor II
LVEF: Left ventricular ejection fraction
GLS: Global longitudinal strain
LA: Left atrium
LV: Left ventricle
HF: Heart failure
PALS: Peak left atrial longitudinal strain
